# A Pilot Study to Propose a "Harm Scale", a New Method to Predict Risk of Harm to the Corneal Endothelium Caused by Longitudinal Phacoemulsification, and the Subsequent Effect of Endothelial Damage on Post Operative Visual Acuity

**DOI:** 10.1371/journal.pone.0146580

**Published:** 2016-01-13

**Authors:** Francesco Saverio Sorrentino, Claudio Bonifazzi, Francesco Parmeggiani, Paolo Perri

**Affiliations:** 1 Department of Surgical Sciences, Unit of Ophthalmology, Ospedale Maggiore, Bologna, Italy; 2 Department of Biomedical and Surgical Sciences, Section of Human Physiology, University of Ferrara, Ferrara, Italy; 3 Department of Biomedical and Surgical Sciences, Division of Ophthalmology, University of Ferrara, Ferrara, Italy; National Eye Institute, UNITED STATES

## Abstract

**Purpose:**

To study the effect of longitudinal phacoemulsification energy on corneal endothelium and to evaluate the relationship between changes of endothelial cells and postoperative visual acuity.

**Methods:**

This prospective clinical observational cohort study included 50 patients with cataract who underwent longitudinal phacoemulsification. Sequential quantitative and qualitative morphometric endothelial cell analyses of the cornea were performed 4 weeks preoperatively and 6 weeks postoperatively using noncontact specular microscopy.

**Results:**

There was a relationship between ECL percentage (ECL%) and the 5-score harm scale, well-described by a linear model (one-way ANOVA, R^2^ = 73.3%). Analyzing the distribution of ECL% Mean with Tukey post-hoc pairwise comparison test (P < 0.001), the value of ECL = 20% has been regarded as cut-off to discriminate patients who obtained an excellent postoperative best-corrected visual acuity (BCVA > 85 letters) from those who just had a good visual outcome (BCVA ≤ 85 letters). There was a significant correlation among the 5-score harm scale, phaco energy intraoperatively delivered, and average cell area postoperatively.

**Conclusions:**

The 5-score harm scale, a new method that enables to pigeonhole cataracts taking into account nucleus hardness and phaco times, allows to predict the harm on corneal endothelium after longitudinal phacoemulsification. Assessment of ECL% permits to discriminate between patients with excellent BCVA and with just good BCVA, postoperatively.

## Introduction

In last two decades, cataract surgery has been markedly improved by means of novel techniques, new surgical equipment and viscoelastic agents [1]. Well-established phacoemulsification techniques and well-experienced surgeons have done their utmost intraoperatively to preserve the anatomical structure of the anterior chamber [2,3]. The care of corneal endothelium has always been one of the main concerns of surgeons during phacoemulsification [4–6]. In fact, mechanical factors such as the jackhammer effect of longitudinal phaco cutting or the implosion of microcavitation air bubbles cause heating and increased high pressure in the anterior chamber [7,8]. Likewise, the ultrasonic power fluctuation, bouncing of fragments, the fluid turbulence and increased production of free radical oxygen species cause great stress on endothelium [9,10].

The corneal endothelium, which is derived from the neural crest, is a single layer of cells that line the posterior corneal surface. Usually, the endothelial cells are hexagonal in shape and uniform in size [11]. Throughout life the central endothelial cell density gradually decreases at an average rate of about 0.6%/year [12], going approximately from 3400 cells/mm^2^ at age of 15 to 2300 cells/mm^2^ at age of 85 years [13]. Under normal circumstances, corneal endothelial cells do not proliferate because they are stuck in the G1 phase of the cell cycle [14,15]. In case of damage to the endothelium, for instance in corneal dystrophies or after intraocular surgery, loss of endothelial cells results in the surviving cells changing in size and shape, becoming large irregular shaped cells to cover the area previously occupied by the lost cells [16]. Acting as a barrier and having a “pump-leak” function, the healthy endothelium is able to maintain the corneal transparency and a well-balanced stromal hydration [17]. Non-contact specular and confocal microscopy are extremely useful devices to clinically investigate and to easily give a morphological evaluation of the corneal endothelium [18].

The purpose of this study was to investigate the effect of energy delivered into the anterior chamber and analyze changes and injury on corneal endothelial cells after longitudinal phacoemulsification. We have used statistical criteria to evaluate the association between phaco times and endothelial damage. To better clarify this correlation, we constructed a Likert-type scale to objectively predict the harm on corneal endothelium. Also, we investigated the relationships between quantitative and qualitative changes of endothelial cells and postoperative visual outcomes.

## Materials and Methods

This prospective longitudinal study included a total of 50 eyes of 50 patients and was conducted from June 2014 to February 2015 at Ferrara University Hospital. The tenets of the Declaration of Helsinki was followed. Institutional review board or ethics committee approval was obtained for each participating institution, and written informed consent from each subject was obtained. The study was in compliance with the Health Insurance Portability and Accountability Act (HIPAA) requirements and was approved by the Institutional Review Board of the Azienda Ospedaliero-Universitaria ‘‘S. Anna”, Ferrara, Italy.

The number of 50 patients with uncomplicated cataract was considered as representative sample of a population that reflects the features of people affected by uncomplicated cataract. Patients were diagnosed with age-related cataract and provided with informed consent. Afterwards, they underwent surgery which was performed by an experienced surgeon. These patients were examined 4 weeks before cataract surgery and followed for 6 weeks after surgery. Exclusion criteria were diabetes and any ocular comorbidities, such as pseudoexfoliation, corneal distrophies, ocular trauma, glaucoma, optic neuropathies, uveitis, high-degree myopia (axial length longer than 26 mm), high-degree hyperopia (axial length shorter than 21 mm), previous intraocular surgery. Patients had a standard ophthalmic exam 4 weeks before surgery and they were scheduled to be seen at 1 day, 5 days and 6 weeks after surgery. The general eye examination, both preoperatively and postoperatively, included slit lamp exam, retinal examination, measure of visual acuity, intraocular pressure, endothelial cell density (ECD), average endothelial cell area (AVG), percentage of hexagonal cells and central corneal thickness (CCT). At slit lamp examination, the nucleus density grade was evaluated according to the Lens Opacities Classification System II (LOCS II) [19]. The non-contact specular microscopy was performed with EM-3000 (Tomey GmbH, Erlangen, D) 4 weeks before and 6 weeks after surgery, in order to measure ECD, AVG, hexagonality, CCT, and to determine the surgically-induced endothelial cell loss (ECL: difference between preoperative ECD and postoperative ECD) [20]. The device is equipped with an autofocus, digital image-capturing system, and automated image analysis software. A fixed area of 0.135 mm^2^ (0.25x0.54 mm) allows for counting up to 300 cells per image. An automated cell contour recognition algorithm, based on contrast differences and area-based counting technique, is used to acquire measurements, with internal calibration for magnification [21]. The phaco machine was the Optikon R-Evolution (Optikon 2000 spa, Rome, IT). The phacoemulsification energy through the phaco needle was set up with longitudinal energy mode. Best-corrected visual acuity (BCVA) was measured at 6-week follow-up postoperatively, using the 4 meter 2000 series revised ETDRS Chart (Precision Vision®). Intraocular lens power was calculated with Sanders-Retzlaff-Kraff/T formula using preoperative keratometry and axial length measurements acquired with IOLMaster 500 (Carl Zeiss Meditec AG, Jena, D). The experienced surgeon did cataract surgery performing the divide and conquer nucleofractis technique and using discovisc (Alcon Laboratories, Inc, Fort Worth, Tex, US) as ophthalmic viscosurgical device [22]. In order to protect the endothelium from undue harm, hydrodissection of the lens cortex and hydrodelineation of the nucleus were performed prior to phacofragmentation. A preloaded hydrophilic single-piece intraocular lens was implanted in the capsular bag for each patient. The surgeon closed the temporal corneal wound with 10.0 nylon suture, removed three weeks after surgery. There were no significant intraoperative complications such as posterior capsule tear, vitreous loss, capsular bag dislocation, or aphakia. Also, there were no significant postoperative complications such as endophthalmitis or severe intraocular inflammation. We used dexamethasone 0.1% eyedrops four times per day for 15 days and nepafenac 0.1% three times per day for 30 days to control postoperative inflammation.

Intraoperatively, we measured the time of phase 1 and 2 of phacoemulsification as well as the total operating time in seconds. Phaco#1 was the time required for cracking of nucleus, Phaco#2 was the time used for fragmentation and aspiration of the four quadrants of nucleus. The continuous mode is a type of power modulation enabling linear control of phaco power by foot pedal [23]. The maximum ultrasound power for each phase was preset by surgeon. Generally, sculpting requires higher energy power and less time than quadrant removal [24]. The machine was set up for phase 1 as follows: ultrasound energy power up to 35%, 30 cmH_2_O for irrigation, 30 mmHg for aspiration. The setting for phase 2 was as follows: ultrasound energy power up to 25%, 110 cmH2O for irrigation, 350 mmHg for aspiration. In our study, Phaco#1 took a relatively short time (from 9 to 43 seconds), while Phaco#2 lasted longer (from 12 to 95 seconds) [24].

We established a new method to describe different aspects involved in the damage and/or loss of endothelial cells after phacoemulsification. For this purpose, we introduced a Likert-type scale, which we called “harm scale”, featured by a score from 1 to 5, covering the range from the lowest level of damage against corneal endothelium to the highest after longitudinal phacoemulsification. Three parameters have been taken into consideration to construct the harm scale. The first was the grade of hardness of cataract evaluated according to the LOCS II. This is a subjective measurement and therefore may not be precise but involve some variability. Conversely, the second and the third parameter were well-defined values because they were objectively measured during surgery. We respectively named the intraoperatively phaco times as Phaco#1 (maximum preset energy power of 35%) and Phaco#2 (maximum preset energy power of 25%). Our goal was using a score to predict ECL% and, consequently, to discriminate between excellent and good postoperative BCVA. All data we collected pre-operatively, intraoperatively and post-operatively can be viewed in Supporting Information (S1 File).

Data were analyzed with the MINITAB 17 software (MINITAB Inc., Pennsylvania State College, USA). A descriptive analysis was performed and the normality test of the data was carried out. The T for student test was used to compare the results between two groups and variance analysis (ANOVA) was used to compare the five levels of the endothelial cell damage. The value of P < 0.05 was considered for statistical significance.

## Results

**Fig 1:**
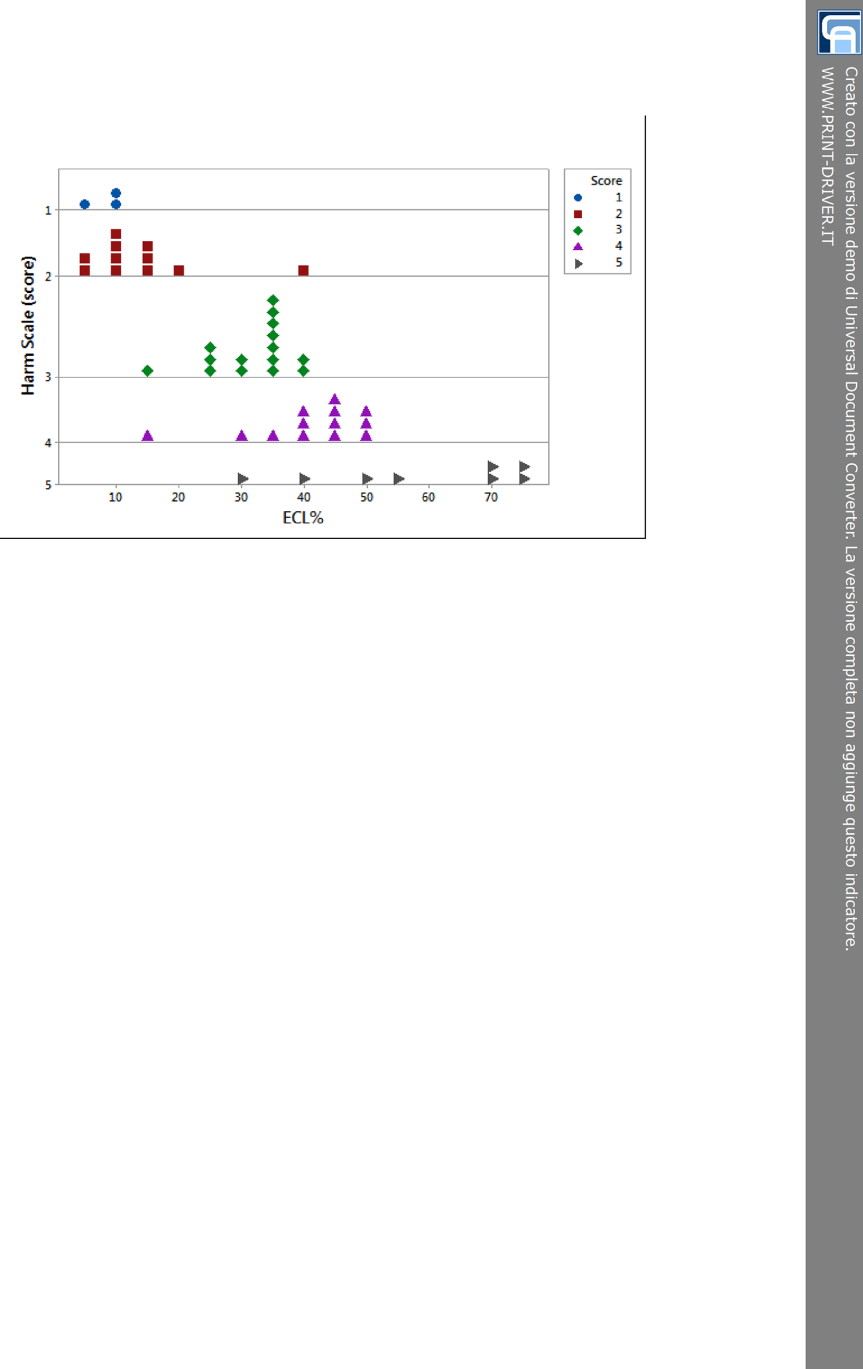
Dotplot of distribution of endothelial cell loss percent within the 5 levels of the harm scale. There are 3 patients in level 1 (blu circle dots), 11 in 2 (red square dots), 15 in 3 (green rhombus dots), 13 in 4 (purple triangle dots), and 8 in 5 (grey right arrow dots). ECL = endothelial cell loss.

The dotplot chart (Fig 1) shows the relationship between ECL% and each score of the harm scale. The distribution of dots efficiently described the daily routine displaying the most of patients in middle scores (from 2 to 4), since very soft and very hard cataracts were less common. The whole level 1 and the most part of 2 overlapped around low percentages of ECL (about 10%). Two conditions could account for that. First, both levels were made up of soft cataracts. Secondly, the sample size in level 1 of the harm scale was small. So we might also state that score 1 and 2 were likely to be merged. The 3-score dots were grouped, somewhat, around values of 30–35% of ECL. The 4-score dots were grouped around 40–45% of ECL, even though some scattered lower values could be detected. The distribution of the 5-score dots was rather dispersed ranging from about 30% to more than 70% of ECL. This shows that an initial evaluation of mature cataract with high LOCS II grading was very likely to predict increased damage on endothelial, although it did not necessarily happen.

**Fig 2:**
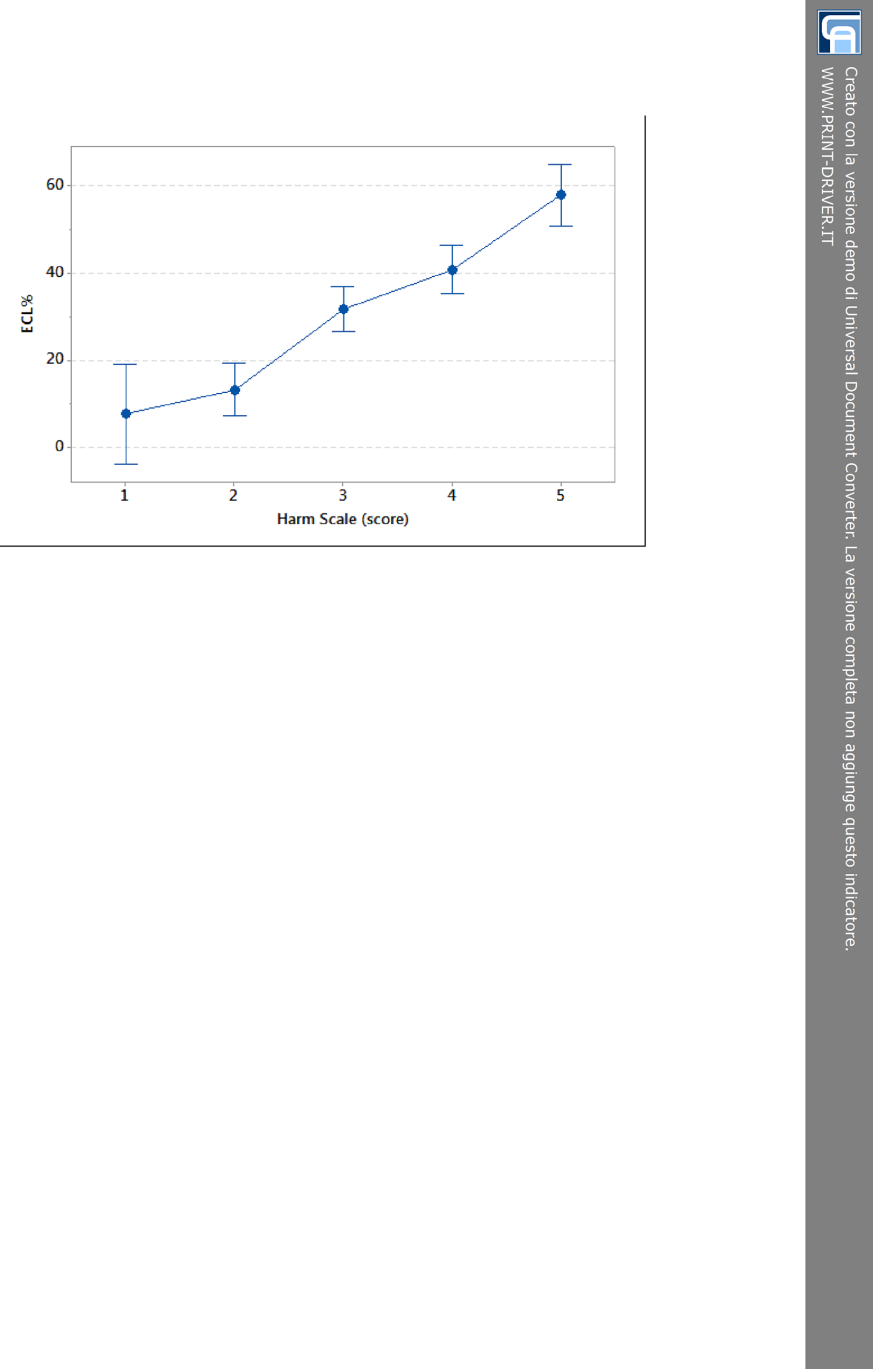
Interval plot of endothelial cell loss resulting from one-way ANOVA test for increasing score values. Tukey post-hoc test groups score 1–2 into subset I, score 3–4 into subset II and score 5 into subset III. The cut-off ECL = 20% splits low scores (subset I) from high scores (subsets II and III). ECL = endothelial cell loss.

The analysis of variance (one-way ANOVA at α = 0.05 level) showed that at different levels of the harm scale ECL% values were grouped according to their dispersion as stated from the partially overlapping interval plots 95% confidence interval (CI) (Tukey post-hoc test, P < 0.001). The linear model displayed in Fig 2 highlights the rising trend of loss of corneal endothelial cells as the score of the harm scale gradually increases (R^2^ = 73.3%). The three subgroups resulting from Tukey post-hoc test confirmed the distribution of ECL% for rising score (Fig 1). The overlap of 95% CI for score 1 and 2 was due to the small sample size. Except for score 1, each interval plot 95% CI for ECL% was about 10% (Fig 2).

**Fig 3:**
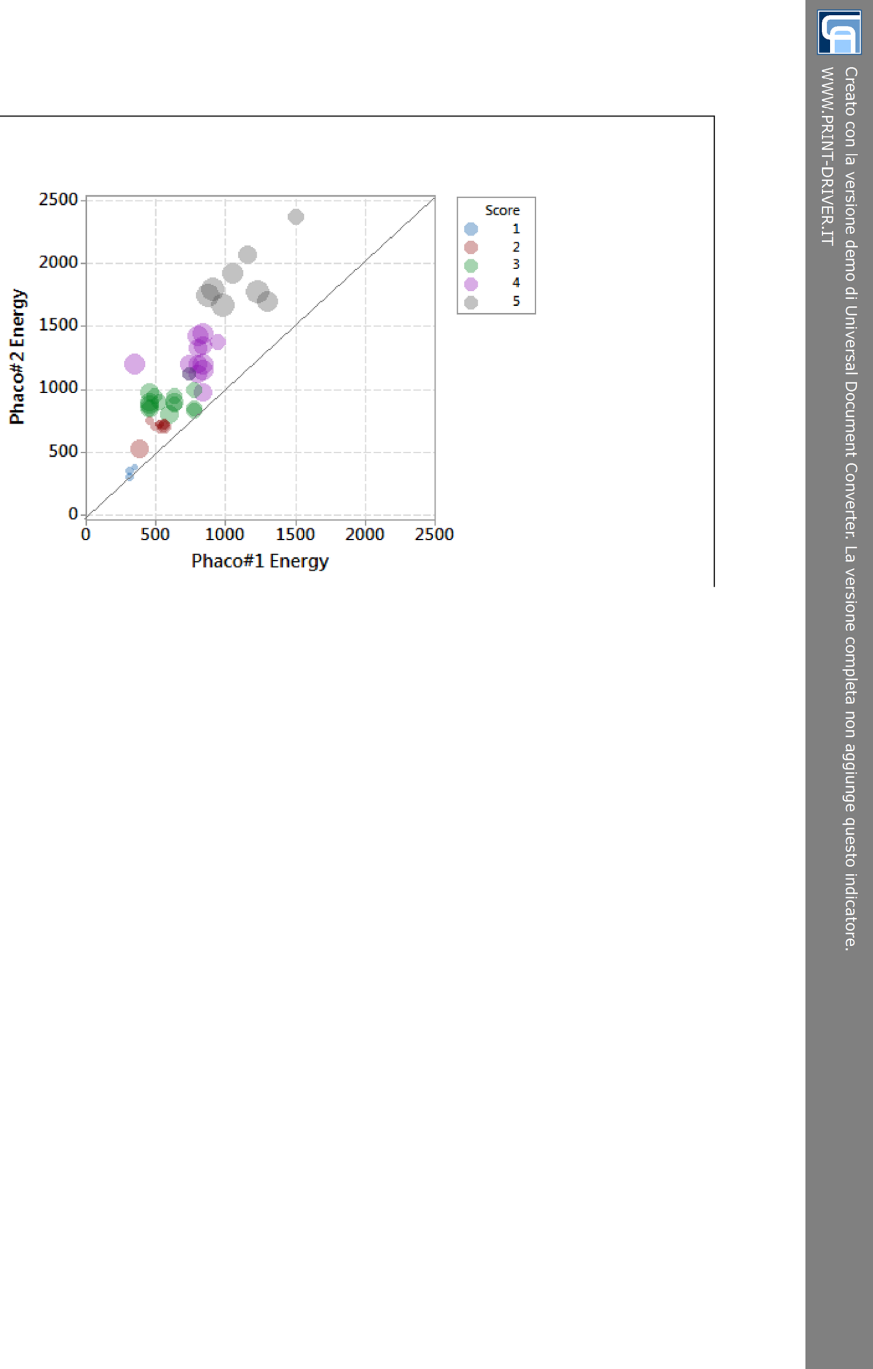
Energy plot of Phaco#1 and Phaco#2 showing the percentage of endothelial cell loss for each score of the harm scale. Small bubbles (light blue and red) for low scores and a progressive boost in bubble size (green, purple and grey) for high scores. Phaco#1 = phaco time to do sculpting; Phaco#2 = phaco time to do quadrant removal; ECL = endothelial cell loss.

The 5-score harm scale has also been used to analyze energy delivered during phacoemulsification. In bubble graph of Fig 3, we named the energy produced during sculpting as Phaco#1 Energy and the one used during quadrant removal as Phaco#2 Energy. There was a positive correlation between the two energy-delivering phases of phacoemulsification. Each bubble has two important features: color and diameter. The first corresponds to the score of the harm scale and the second to ECL%. In other words, colors have been used to categorize the 50 cataracts according to the 5-score harm scale (Fig 1), whereas the width of bubbles indicated the loss of endothelial cells. The narrower the diameter, the lower ECL% occurred. The larger the bubble size, the higher ECL% took place. The distinct bubbles, correlating with specific values of Phaco#1 Energy and Phaco#2 Energy, spread out asymmetrically over the bisector of the scatterplot (Fig 3). The bisector indicates values of equal energy and splits the plane: almost all bubbles lie over the up left half-plane, where values of Phaco#2 Energy are higher than matching values of Phaco#1 Energy. Therefore, ECL% (bubble size) is affected by both times (Phaco#1 and Phaco#2), that is by the total amount of energy delivered into the anterior chamber, which is significantly higher during Phaco#2 (Fig 3).

**Fig 4:**
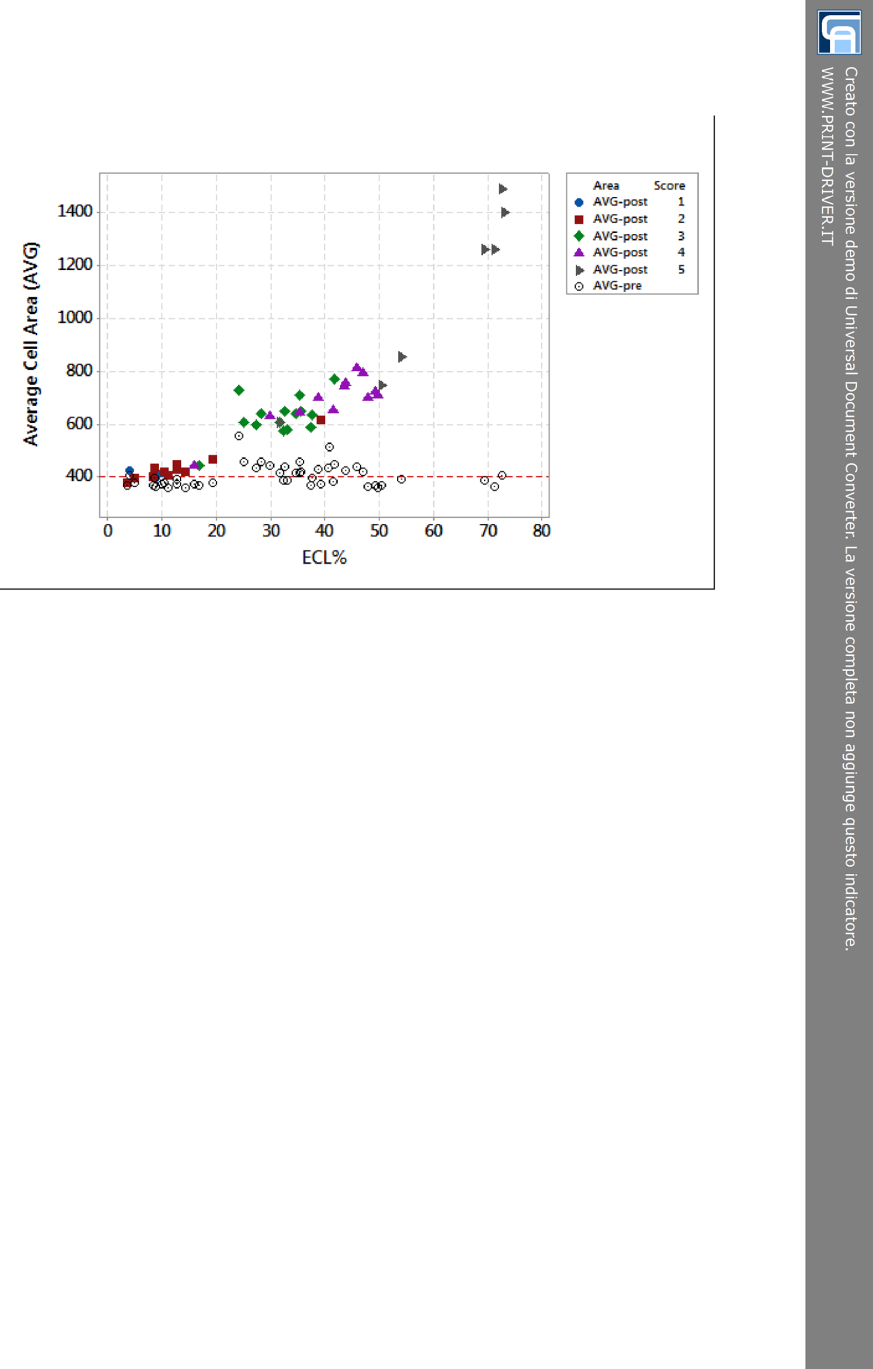
The relationship between average cell area and percentage of endothelial cell loss depending on the 5-score harm scale. The line drawn at 400 μm^2^ highlights the mean value of AVG before surgery. All preoperative values (AVG-pre) are depicted as empty circle dots. Colored dots correspond to postoperative values (AVG-post). AVG-post 1 (blue circle dots) and 2 (red square dots) lie on the bottom left of dotplot corresponding to AVG average of 450 μm^2^ and ECL less than 20%. AVG-post 3 (green rhombus dots) and 4 (purple triangle dots) stay between values of 600 and 800 μm^2^ equivalent to ECL ranging from 25% to 50%. AVG-post 5 (grey right arrow dots) spread out on the up right featured by AVG from 750 to 1500 μm^2^ and ECL from 50% to more than 70%. AVG = average cell area. ECL = endothelial cell loss; AVG-pre = preoperative average cell area; AVG-post = postoperative average cell area.

Also, we focused on significant changes in polymegatism after cataract surgery following modifications in AVG. The dotplot of Fig 4 shows the frequency distribution of AVG for each score of the harm scale depending on ECL%. We have chosen not to graphically differentiate preoperative values of AVG because they all were rather overlapping. But we have used specific types and colors to mark dots belonging to the same level of the harm scale after surgery. It was interesting to observe the rising trend of AVG for increasing score. There was a sort of flattening for low scores (AVG-post 1 and 2 dots) close to 400 μm^2^. It gradually moved upwards for intermediate scores (AVG-post 3 and 4 dots) and it steeply went up for the highest score (AVG-post 5 dots). For rising score, the trend showed an exponential increase of postoperative AVG, particularly after values matched to ECL ≥ 20%. This trend was well-described by a second degree regression equation (P < 0.001, R^2^ = 93%).

**Fig 5:**
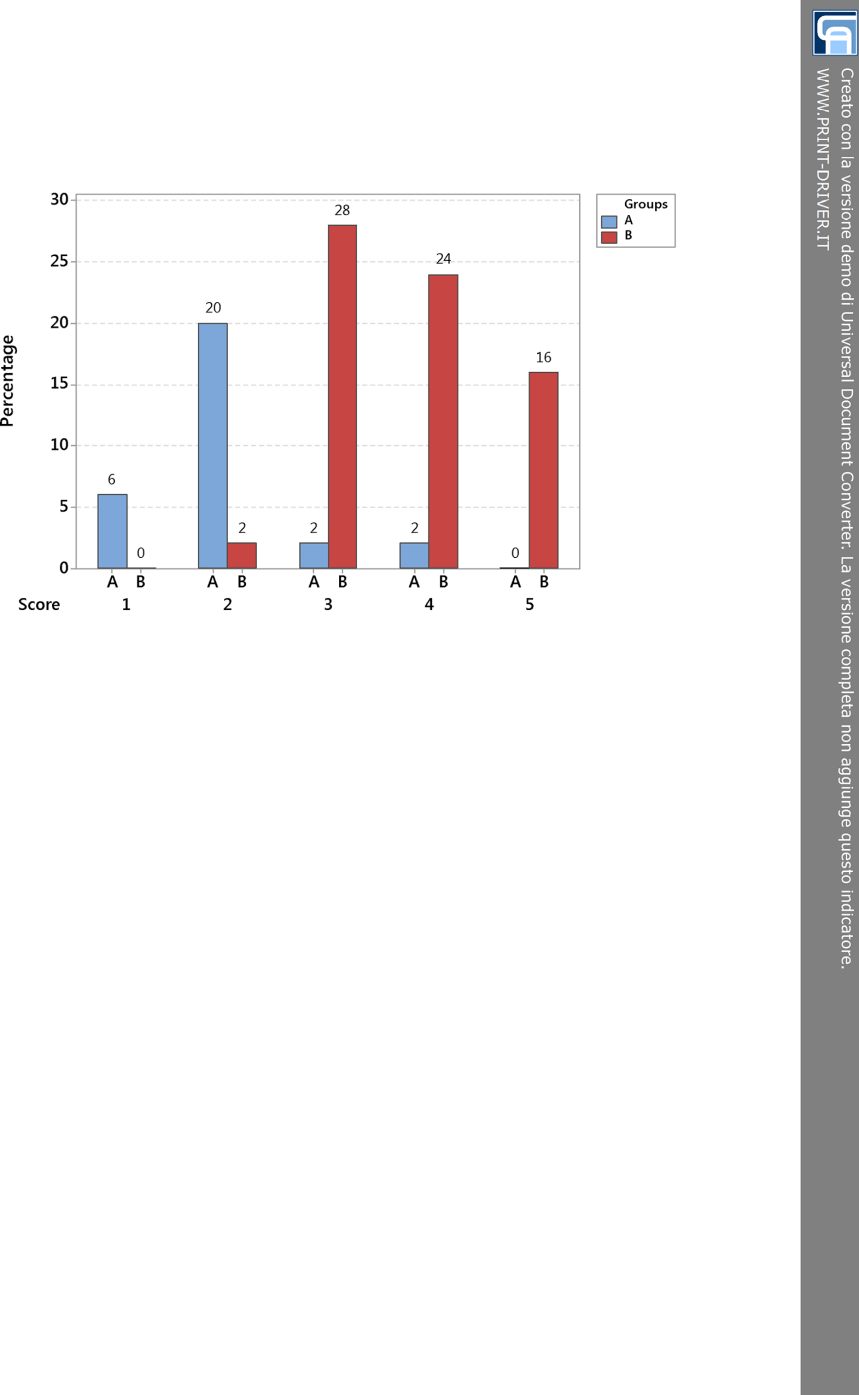
Bar chart compares group A (ECL < 20%) and B (ECL ≥ 20%) for each level of the 5-score harm scale. The cut-off value of ECL = 20% results from Tukey post-hoc test, which states a distinction between scores 1–2 and 3-4-5 (see 95% confidence interval in Fig 2). For rising scores, there is a boost in percentage of people of group B and a relevant drop in percentage of people of group A. ECL = endothelial cell loss.

After describing the relationships among phaco energy, ECL%, AVG-post (postoperative AVG) and 5-score harm scale, we analyzed two groups of patients, which we named A and B, on the strength of cut-off value of ECL = 20% as above stated by the one-way ANOVA (Fig 2). We chose this cut-off value to draw a discrimination between patients who were likely to have an excellent recovery of visual acuity after surgery (more than 85 letters of ETDRS) and patients who were going to have just a good recovery of visual acuity (85 letters of ETDRS or less). The group A was characterized by ECL less than 20%, whilst the group B included patients with ECL equal to or more than 20%. After cataract surgery, there were more patients in group B (36 people, 70% of sample) than A (14 people, 30% of sample). In fact, the number of people with cataract of score 3 or higher was larger and most of them were characterized by a quite remarkable damage and/or loss of endothelial cells. Comparing group A and B (Fig 5) for each level of the harm scale, it was notable that group A was well-represented for low scores and less depicted for high scores till to be unrepresented in score 5, whilst group B was not displayed in score 1 and progressively well-represented in the following scores till to be the only group in level 5.

**Fig 6:**
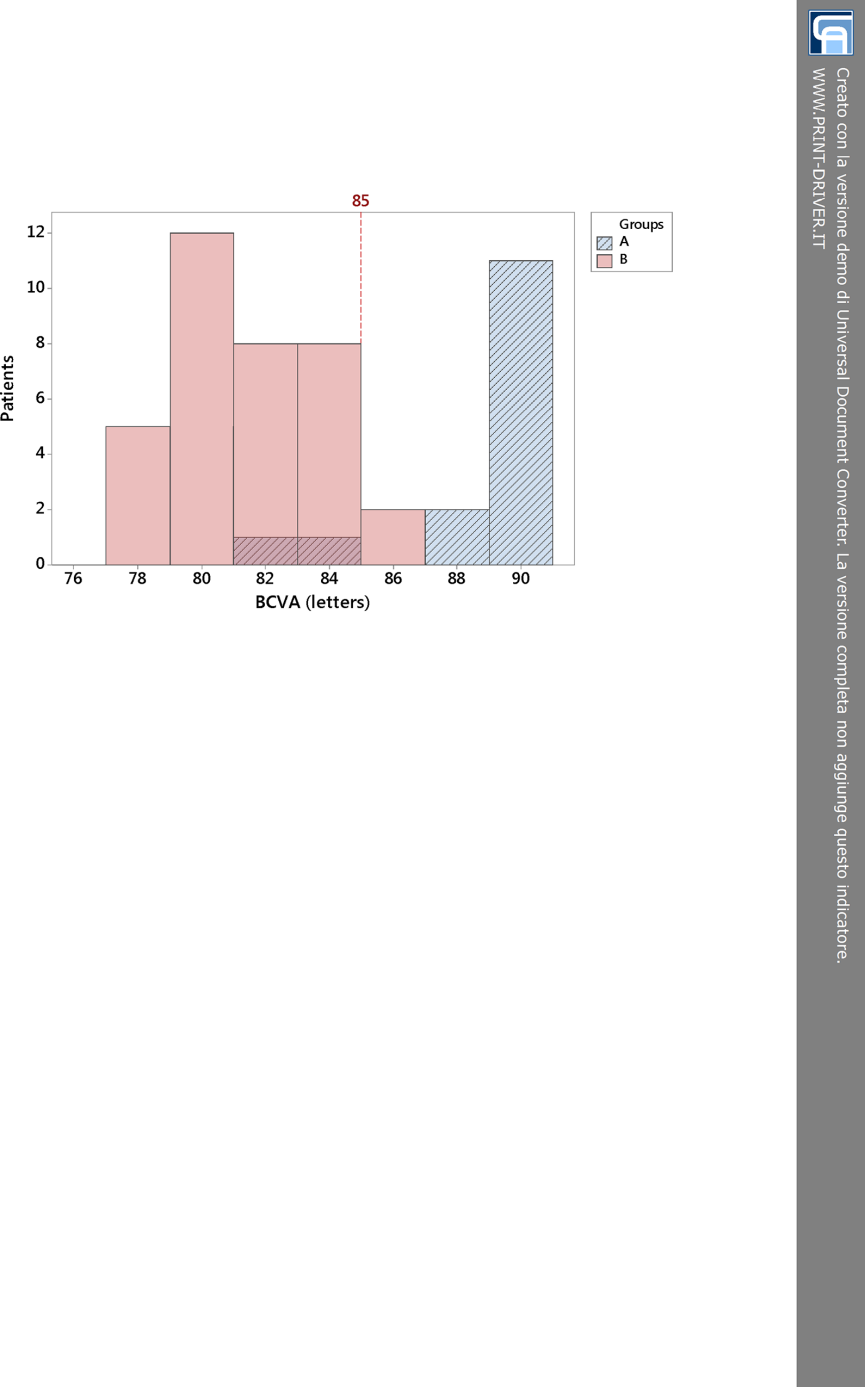
Frequency distribution of postoperative best-corrected visual acuity in group A (ECL < 20%) and B (ECL ≥ 20%) at 6-week follow-up. Line at 85 letters discriminates an outstanding visual recovery (group A) from just a good visual outcome (group B). BCVA = best-corrected visual acuity; ECL = endothelial cell loss.

Starting from the relevant differences observed in ECL% between groups A and B for increasing score values, we decided to analyze and compare BCVAs of patients in both groups, evaluating how the loss of endothelial cells due to longitudinal phacoemulsification could affect the visual acuity at 6-week follow-up. The histogram in Fig 6 shows the comparison between BCVA of group A (ECL < 20%) and group B (ECL ≥ 20%). Both groups had quite a good recovery of visual function, but there was a substantial difference between them. Fixing a cut-off at 85 letters, which meant a good visual outcome after phacoemulsification, we could see that almost 85% of people in group A had a visual acuity letter score better than 85 at ETDRS chart, whereas less than 10% of group B had the same result. By contrast, approximately 15% of subjects in group A and more than 90% in group B had BCVA less than 85 letters postoperatively.

## Discussion

In our study, we established a new method to pigeonhole different cataracts according to hardness and phaco times. Our primary end-point was to make a connection between any type of cataract and the harm on corneal endothelium after longitudinal phacoemulsification. Our secondary end-point was to establish a link between loss of corneal endothelial cells and visual outcome postoperatively. To do that, we constructed a Likert-type scale giving a score from 1 to 5 to each cataract (Table 1), summarizing the hardness grading and the phaco times including sculpting (Phaco#1) and quadrant removal (Phaco#2), both intraoperatively acquired. We named this scale as “harm scale” to highlight changes and/or damage on corneal endothelium [25–27]. We considered to maintain the distinction in five levels because, with larger samples, the score 1 could be the fittest for soft cataracts whilst the score 5 for very hard cataracts. Hence, we have used the score to evaluate different aspects related to phacoemulsification. We also stated that hardness grading was a preliminary approximate evaluation of any cataract and it could just provide vague indications for surgical procedure. In fact, doing surgery as well as managing complications, mainly for cataracts more complex than preliminary expected, feature a certain intraoperative variability. Therefore, the harm scale might be regarded as a sort of refinement of the initial assessment of hardness, also comprising intraoperative phaco times.

**Table 1:** The construction of the harm scale.

Score of Likert-type harm scale	1	2	3	4	5
Hardness	1	1–2	2–3	3–4	4
Phaco#1	<10	<16	<22	<28	≥28
Phaco#2	<15	<30	<45	<60	≥60
Number of patients	3	11	15	13	8
ECL% Mean	7.64	13.30	31.66	40.82	57.99
ECL% SE	1.82	2.91	1.64	2.61	5.68
AVG Mean	409.7	436.1	626.9	691.4	1088.0
AVG SE	7.7	19.3	19.6	27.7	131.0

Phaco#1, phaco time to do sculpting; Phaco#2, phaco time to do quadrant removal; ECL, endothelial cell loss; SE, standard error; AVG, average cell area. The upper part shows the method of construction of the Likert-type harm scale by collecting three parameters (hardness grading, Phaco#1 and Phaco#2) and partitioning into 5 scores. Hardness grading depends on Lens Opacities Classification System II; Phaco#1 and Phaco#2 are times measured in seconds. The bottom part displays the rising trends of endothelial cell loss and average cell area for increasing score; ECL% and AVG standard errors give indications of the accuracy of such analysis.

First of all, we confirmed that damage on endothelial cells is due to both phases of phacoemulsification, particularly to the second one. Less powerful ultrasounds but for longer time trigger higher delivered energy into the anterior chamber and, consequently, endothelial cell loss. We established a connection between phaco energy and percentage of endothelial cell loss (ECL%) according to the 5-score harm scale (Fig 3). In our investigation, we didn’t analyze the effects of fluidics and vacuum, which are supposedly time-dependent changing between Phaco#1 and Phaco#2 significantly [27]. New research would be to conduct to try to establish connections among fluidics, vacuum, energy and final harm on corneal endothelium.

Table 2 sums up our results and considerations. It shows that ECL% and AVG-post progressively rose for increasing scores. Furthermore, the five levels could be grouped in 3 subsets as certified by Tukey post-hoc test (P < 0.001): grouping I consisted of score 1 and 2, grouping II of score 3 and 4, grouping III of score 5. These outcomes enabled us to establish the 20% of ECL as cut-off to discriminate low score (subset I) from high score (subsets II and III) values. Hence, we used this result to obtain two ECL groups (A for subset I, B for subsets II and III) to mark the difference in BCVA postoperatively. We noticed that patients having ECL less than 20% could get an excellent visual recovery measured as postoperative BCVA > 85 letters, whereas patients with ECL greater or equal 20% could get just a good visual acuity postoperatively, yet with a BCVA ≤ 85 letters.

**Table 2:** An overview of the 5-score harm scale: significant correlations.

Score	Patients	Post-Hoc Tukey Groups	ECL % Mean	AVG-post Mean	BCVA > 85 Letters	BCVA ≤ 85 Letters
1	3	I	7.6	409.7	3	0
2	11	I	13.3	436.1	10	1
3	15	II	31.6	626.9	1	14
4	13	II	40.8	691.4	1	12
5	8	III	58.0	1088.0	0	8

ECL, endothelial cell loss; AVG-post, postoperative average cell area; BCVA, best-corrected visual acuity. Mean values of percentage of endothelial cell loss and postoperative average cell area related to the 5-score harm scale as results from post-hoc Tukey test. The last two columns highlight the number of patients who obtained an excellent best-corrected visual acuity (BCVA > 85 letters) and just a good visual outcome (BCVA ≤ 85 letters) postoperatively.

As considered in our results, dynamic changes in morphometric parameters (polymegathism and pleomorphism) might be sensitive to predict endothelial recovery after cataract surgery [28,29]. Analyzing AVG postoperatively, we could appreciate that it reproduced the results of Tukey post-hoc test. Comparing the trends of ECL% Mean, AVG-post Mean and the subsets displayed in Table 2, we observed a strong correlation. As the loss of endothelial cells took place, the “surviving” cells enlarged their area occupying as much space as they could. Moreover, the relationship between AVG-post and ECL% (Fig 4) showed a discrimination between low scores (1–2, group A) and high scores (3-4-5, group B). Considering that the preoperative mean AVG was about 400 μm^2^, we noticed that group A was characterized by AVG-post just slightly augmented, while group B had AVG-post significantly and progressively enlarged. Therefore, small changes in endothelial cell sizes after surgery (AVG-post for group A) were related to an excellent visual outcome (BCVA > 85 letters), whereas sizeable changes (AVG-post for group B) correlated with just a good visual acuity postoperatively (BCVA ≤ 85 letters).

We have also analyzed hexagonality, reflecting variation in endothelial cell shape, and CCT [28]. In our results, there was little of significant changes for both parameters between 4-week preoperatively and 6-week postoperatively. We used the 5-score harm scale to correlate ECL% with polymorphism. Regardless of the score, there were fluctuations in the percentage of hexagonality and just a slightly downward trend after phacoemulsification (not graphically shown). We can hypothesize that “surviving” endothelial cells counterbalance the missing cells trying to reach a shape as much regular as possible. Anyway, more studies are required to better investigate this aspect. Concerning CCT as indicator of endothelial damage, we just calculated CCT-pre Mean (534.87 μm, StDev 35.78) and CCT-post Mean (535.37 μm, StDev 35.77), but we definitely did not detect any significant modification between preoperatively and postoperatively (t-Test, P = 0.47) [30–32].

Finally, it could be argued that the sample size of our investigation was not very large. Probably, bigger sample size could have given the opportunity to fine-tune the harm scale we constructed, avoiding dispersion of some values in high scores. To our knowledge, this study has been the first that analytically investigated the loss and the actual increase in size of endothelial cells depending on phaco energy. Moreover, it highlighted that there was a link between ECL% and postoperative BCVA, somehow. Anyway, further studies are needed to better manage the use of this new method of analysis.

In conclusion, the harm scale is a good strategy to predict damage on corneal endothelium after longitudinal phacoemulsification as well as quantitative changes in endothelial cell size. The relationship between loss of corneal endothelial cells and postoperative visual acuity is of paramount importance in order to make maximum efforts to reduce phaco energy as much as possible. Certainly, new investigations can apply this objective analysis to other techniques such as phaco-chop or to other phaco energy mode settings such as torsional so as to compare different surgical procedures according to delivered energy into the anterior chamber [33,34].

## Supporting Information

S1 File Data used to construct the harm scale and to analyze corneal endothelium modifications after the surgical procedure of phacoemulsification. (XLS)
